# Moral Disengagement Mechanisms and Personality Dimensions Implicit to Homophobia

**DOI:** 10.3390/ijerph19148583

**Published:** 2022-07-14

**Authors:** José Antonio González-Fuentes, Juan Manuel Moreno-Manso, Mónica Guerrero-Molina, Eloísa Guerrero-Barona, María Elena García-Baamonde

**Affiliations:** Department of Psychology, University of Extremadura, 06006 Badajoz, Spain; jogonzalezf@unex.es (J.A.G.-F.); monicagm@unex.es (M.G.-M.); eloisa@unex.es (E.G.-B.); mgarsan@unex.es (M.E.G.-B.)

**Keywords:** homophobia, moral disengagement, personality, sexual discrimination, youth

## Abstract

Recent advances in sexual equality and diversity have not been able to mitigate the serious problem of discrimination suffered by sexual minorities. The most serious cases involve violence and physical or psychological aggression towards sexual orientations that differ from the heterosexual norm. This research analyses the dimensions of the personality and the moral disengagement mechanisms related to homophobia and the predictive value they have for hostile attitudes towards sexual diversity. The sample was made up of 849 university students between 18 and 24 years of age. The instruments used were the Modern Homophobia Scale (MHS), the Mechanisms of Moral Disengagement Scale (MMDS), and the reduced version of the Neo Personality Inventory—Reduced Version (NEO-FFI). The results show the involvement of moral disengagement in homophobia. It highlights evidence of subtle intimidatory behaviour patterns of rejection towards homosexuality. Furthermore, the low levels in the dimensions of a friendly personality and openness to experiences can be seen to predict homophobic behaviour. Thus, young people fall back on diverse mechanisms of moral disengagement to justify harmful attitudes towards the LGTBI collective. The results of the research are particularly relevant and useful for setting up programmes aimed at preventing and mitigating this serious problem of sexual discrimination.

## 1. Introduction

Despite the advances made concerning sexual diversity and equality, in today’s society, homosexuality is still a stigma that excludes and discriminates against persons because of their sexual orientation. In this sense, homophobia is considered a tendency to be rejected, either subtly or directly, by both gays and lesbians, because of their deviation from the heterosexual norm, giving rise to fear and uncomfortable situations in the presence of this collective which, in the most serious cases, can lead to psychological and physical aggression and violence [1,2]. 

In Spain, the data from the Interior Ministry [3,4], in its report concerning the evolution of hate crimes against the LGTBI collective, show an increase of 8% in cases over the period 2018 to 2020. The data provided by the network’s observatory against hate [5] show 971 cases over the last year, which supposes a considerable increase, where the most frequent crimes are verbal aggression and insults, followed by harassment and intimidation in over half the cases registered. In addition, 17% ended in physical aggression. 68% of the victims belonged to the age group between 18 and 35 years of age.

Different works of research demonstrate that homophobia is related to certain dimensions of the personality [6] and that they may predispose individuals to form particular types of attitudes, and that in relation to the LGTBI collective, such attitudes appear as specific negative responses towards homosexuality [7]. In this respect, starting from the personality model of the Big Five, widely accepted by the scientific community [8,9], it has been found that homophobia correlates negatively with the personality dimension open to experiences [7,10,11,12]. Thus, the behaviour patterns that are the most conventional, conservative, and “closed” to experiences can be related to a greater intolerance towards the LGTBI collective. Other studies have related the personality dimension neuroticism to homophobic behaviour patterns [12], concluding that the subjects with high scores in this factor concerning personality are more likely to violate the rights of minorities. These homophobic persons can show such traits as emotional instability, irritability, and hostility towards the LGTBI collective. As for the personality dimensions of agreeableness, conscientiousness, and extraversion, Horne et al. [13], Miller et al. [14], and Oltra et al. [12] found a negative correlation with homophobia. Thus, in general terms, a low agreeableness, social sensitivity, assertiveness, conscientiousness, and personal and social responsibility can predict more intolerant, conflictive, irresponsible, aggressive, and offensive behaviour [15,16,17]. 

One variable that is related to the appearance of intimidatory and aggressive behaviour is moral disengagement. The self-control system for moral behaviour can be temporarily deactivated in certain circumstances, causing morally maladapted behaviour patterns concerning that which the person does not feel any scruples [18,19,20]. In this sense, different research works have pointed out that moral disengagement can be a good predictor of aggressive, intimidatory, and antisocial behaviour [21,22,23]. Among the specific mechanisms of moral disengagement, the most common are displacement of responsibility [24], attributing blame, and moral justification [25,26]. 

There are very few works of research aimed at studying moral disengagement in homophobic behaviour patterns, and those few that do exist have a generalist nature, without specifying the mechanisms of moral disengagement that can be part of the hostility towards and rejection of this collective. Carrera-Fernández et al. [27] found high levels of moral disengagement in persons who showed general racist and homophobic attitudes, while Sahlman [28] identified the presence of moral justification mechanisms in harmful communication towards the LGTBI collective.

On the other hand, there are several studies that have connected moral disengagement to some personality dimensions. However, these works of research have not focused specifically on homophobia, and their results have not been consistent. Sagone and De Caroli [29] and Walters [17] found that the personality dimensions of neuroticism, extraversion, and openness to experiences correlated positively with moral disengagement, while agreeableness and conscientiousness did so negatively. Nevertheless, Caprara et al. [30] found that neuroticism and agreeableness were the only dimensions that correlated with moral disengagement. Other studies, such as that of Egan et al. [31], have concluded that scarce agreeableness and conscientiousness correlated with a high level of moral disengagement in the persons. However, they found no association between moral disengagement and extraversion or neuroticism. Similarly, Zhou et al. [32] found that moral disengagement correlated negatively with agreeableness, conscientiousness, and extraversion but positively with neuroticism, while no relation was found between moral disengagement and the factor of openness to experiences. As we have been able to verify, the greatest agreement exists around the fact that the dimensions of agreeableness and conscientiousness are negatively related to moral disengagement, with certain discrepancies being established in this relation for the remaining factors. 

Based on the above, Gini et al. [21] concluded that there is a lack of research into the possible mediating role of moral disengagement in homophobic behaviour patterns. Similarly, considering the disparity in the results concerning the relationship between personality dimensions and moral disengagement [27,28], we raise the question of whether there may be a relation between certain personality dimensions and the mechanisms of moral disengagement that can predict intimidatory and abusive homophobic behaviour. 

In this context, the objectives of this research were: to analyse homophobic attitudes in a sample of Spanish university students; to examine the personality dimensions that are related to homophobic attitudes; to study the existing relation between moral disengagement and homophobia, identifying the commonest mechanisms of moral disengagement; and finally, to determine the predictive value of the personality dimensions and the mechanisms of moral disengagement in homophobic attitudes. Based on the theoretical review carried out, we expected some students to express homophobic attitudes (Hypothesis 1). Furthermore, we expected that such personality dimensions as openness to experiences, agreeableness, extraversion, and conscientiousness would be related to more positive attitudes towards homosexuality, while neuroticism would be more connected to homophobic attitudes (Hypothesis 2). In addition, we also expected that moral disengagement would be related to homophobia in such a way that greater levels of homophobia would mean greater use of moral disengagement mechanisms, the commonest being the transfer and diffusion of responsibility, distortion of consequences, blaming the victim, and moral justification (Hypothesis 3). Finally, we anticipated that certain personality dimensions and moral disengagement mechanisms would act as predictors of the homophobic attitudes of the youths (Hypothesis 4).

## 2. Materials and Methods

### 2.1. Participants 

The sample was made up of 849 youths between 18 and 24 years of age (M = 20.07; DT = 1.74), of whom 58.1% were female (N = 493) and 41.9% were male (N = 356). The sample was made up of students from their final year at the University of Extremadura (Spain), studying different degrees at campuses in Badajoz and Caceres. The research was carried out during the academic year of 2020/2021. The selection of the students was carried out by means of a non-probabilistic sample of convenience so as to select a representative sample of the total student population at the University of Extremadura. The study did not consider the sexual orientation of the participants as a criterion during the sample selection process. The research involved students of different sexual orientations. The participants in the study came from families with a medium socioeconomic level as far as studies, income, and work situation are concerned.

### 2.2. Instruments

1. Modern Homophobia Scale (MHS). This scale by Raja and Stokes [33], in its Spanish adaptation [34], was used to evaluate homophobia. This instrument consists of two subscales: 1. MHS-G is made-up of 22 items that evaluate the degree of acceptance or rejection towards the gay collective; and 2. MSH-L is made-up of 24 items that measure the degree of acceptance or rejection towards the lesbian collective. In addition, each subscale evaluates three attitudes towards the gay/lesbian collectives (personal discomfort, deflection/changeability, and institutional homophobia). The first two factors show attitudes of a personal type towards these collectives, while the third refers to institutional attitudes of acceptance or rejection. The responses are given on a Likert-type scale of 5 points, where 1 means totally disagree, and 5 means totally agree, in which the higher scores are related to homophobic attitudes. As for the psychometric properties of the scale, Raja and Stokes [33] obtained a reliability of α = 0.94 for the subscale MHS-G and α = 0.93 for MSH-L. Furthermore, Rodríguez et al. [34] reported reliability similar to that of the original scale for MHS-G, with α = 0.91 in personal discomfort, α = 0.85 in deflection/changeability, and α = 0.85 in institutional homophobia; and for MHS-L, α = 0.90 in personal discomfort, α = 0.90 in deflection/changeability, and α = 0.83 in institutional homophobia.

2. Mechanisms of Moral Disengagement Scale (MMDS). This scale [18] was used to measure moral disengagement in its Spanish adaptation [35]. This instrument consists of 32 items whose responses are given on a Likert-type scale of 5 points, where 1 means totally disagree, and 5 means totally agree. It is made up of eight subscales that correspond to the mechanisms of moral disengagement (moral justification, euphemistic language, advantageous comparison, transfer of responsibility, diffusion of responsibility, distortion of the consequences, attributing blame, and dehumanisation). Based on these subscales, it is possible to obtain partial scores for each mechanism, as well as a total score. The general reliability of the instrument, according to the various works of research, oscillates between 0.82 and 0.93 of Cronbach’s α [36]. The reliability of the Spanish adaptation obtained similar results of α = 0.87 [35].

3. Neo Personality Inventory—Reduced Version (NEO—FFI) [37,38,39]. This evaluates personality using the revised Spanish version [40]. This instrument consists of 60 items whose responses are given on a Likert-type scale of 5 points, where 1 means totally disagree, and 5 means totally agree. It is made up of five personality factors: openness to experiences (O), responsibility/conscientiousness (C), extraversion (E), agreeability (A), and neuroticism (N). These five factors have been revised and replicated in studies carried out with diverse cultures in different countries [41,42,43], verifying the universality of the NEO—FFI [44]. The original version reached values of high reliability, between α = 0.88 and α = 0.92. The Spanish adaptation reported similar reliability to the original, α = 0.82 for neuroticism, α = 0.81 in extraversion, α = 0.76 in openness to experiences, α = 0.71 in agreeability, and α = 0.80 in responsibility/conscientiousness [45]. 

### 2.3. Procedure

The research was authorised and approved by the University of Extremadura. Prior to applying the measurement instruments, authorisation was requested from the deans and lecturers of the faculties that participated in the research. The students were given an informative sheet explaining the purpose of the study and the confidentiality of the data provided, as well as the voluntary nature of the participation. The participating students also provided informed consent. The researchers applied the instruments collectively, and the duration was around 45 min. The instruments were applied by two evaluators in a single session. There were no difficulties during test administration.

All procedures performed were in accordance with the ethical standards of Extremadura University (Ref.: 181/2020) and with the 1964 Helsinki declaration and its later amendments or comparable ethical standards.

### 2.4. Data Analysis

The data obtained through the evaluation instruments were processed using a statistical package IBM SPSS Statistics v.25. 

We first carried out a descriptive analysis to study the homophobic attitudes of the participants. We then performed parametric tests to check the nature of the variables and the sample size (n = 849). In this sense, a correlation analysis was done using Pearson’s coefficient to analyse the relationship between the mechanisms of moral disengagement, personality traits, and modern homophobia.

Finally, we carried out a linear regression analysis to determine how far the mechanisms of moral disengagement and personality traits can significantly predict attitudes of tolerance towards masculine and feminine homosexuality.

## 3. Results

Table 1 shows the descriptive analysis of the Modern Homophobia Scale (MHS). 

In general, the results demonstrate that the university students have positive attitudes towards both masculine (M = 1.97; SD = 1.09) and feminine (M = 1.94; SD = 1.05) homosexuality. Nevertheless, lower scores were obtained in the total scales of homophobia for lesbians and in the subscales of personal discomfort (lesbians) and deflection/changeability (lesbians), indicating that there exists a greater tolerance towards feminine homosexuality as opposed to masculine sexuality. 

As for the subscale institutional homophobia, it is interesting to note that the students considered that the institutional practices and policies ought to be as free as possible from bias as far as gays (M = 2.12; SD = 1.15) and lesbians (M = 2.43; SD = 1.16) are concerned.

Below, Table 2 shows the results of the correlation analysis to check whether modern homophobia is related to moral disengagement and personality dimensions.

The results show for the students a positive correlation between moral disengagement and the subscales of modern homophobia (*p* < 0.001). This supposes that the greater the use of the mechanisms which allow one to legitimise acts against the moral system, the greater the homophobia. Thus, the relation between moral disengagement and the necessity to avoid personal contact with gays and lesbians becomes apparent, as well as the belief that gays and lesbians can change their sexual orientation at will and the consideration that institutional policies are not free from bias as far as persons’ sexual orientation is concerned. 

As for the subscales of moral disengagement, we can see that modern homophobia is related to moral justification (*p* < 0.001), euphemistic language (*p* < 0.001), advantageous comparison (*p* < 0.001), transfer of responsibility (*p* < 0.001), diffusion of responsibility (*p* < 0.001), distortion of consequences (*p* < 0.001), dehumanisation (*p* < 0.001), and attributing blame (*p* < 0.001). Thus, the lower the tolerance towards masculine and feminine homosexuality, the greater the tendency to justify harmful behaviour patterns as a means to achieving superior moral purposes or values; to reduce or misrepresent immoral behaviour through language; to make comparisons between one’s own conduct and that of others, which is considered to be worse; to attribute the responsibility of one’s own actions to other persons or situations; to spread the responsibility among the group participating in the immoral conduct; to distort or minimise the consequences of the immoral actions; to play down the harm done to those affected by the damaging behaviour, and to make the victim the one mainly responsible for the immoral conduct. 

As for the personality dimensions, the data indicate that there exists a negative correlation between agreeableness and all the subscales of modern homophobia (*p* < 0.001) so that being compassionate and sensitive and being willing to avoid conflicts are related to positive attitudes toward gays and lesbians. Similarly, we found that openness to experiences correlates negatively with the subscales of personal discomfort concerning gays (*p* = 0.015) and the total scale of homophobia concerning gays (*p* = 0.046). This would indicate that being open to experiences is related to a greater tolerance towards masculine homosexuality and a lesser feeling of discomfort in the presence of gay couples. Finally, the results show a negative correlation between the factor of conscientiousness/responsibility and the dimension of deflection/changeability concerning lesbians (*p* = 0.025); so, being responsible and organised and having solid principles are related to a lesser tendency to consider that lesbian women can change their sexual orientation at will. 

To conclude, we carried out a linear regression analysis to check whether moral disengagement and personality dimensions can predict modern homophobic attitudes towards the gay collective (Table 3) and the lesbian collective (Table 4). 

The results indicate that moral disengagement can significantly predict negative attitudes towards gays and lesbians. To be precise, moral disengagement in these students explains 32.9% of the variance in their responses in personal discomfort concerning gays (*p* < 0.001), 24% in deflection/changeability concerning gays (*p* < 0.001), 32.8% in institutional homophobia concerning gays (*p* < 0.001), 36.6% in homophobia concerning gays (*p* < 0.001), 31.2% in personal discomfort concerning lesbians (*p* < 0.001), 24.7% in deflection/changeability concerning lesbians (*p* < 0.001), 25.8% in institutional homophobia concerning lesbians (*p* < 0.001), and 34.1% in homophobia concerning lesbians (*p* < 0.001). In this sense, it can be said that all the mechanisms of moral disengagement that were analysed predict a lower tolerance towards gays and lesbians. 

As for the personality dimensions, the results indicate that agreeableness predicts tolerant attitudes towards gays and lesbians. Agreeableness predicts 2.2% of the variability of the responses in personal discomfort concerning gays (*p* < 0.001), 1.5% in deflection/changeability concerning gays (*p* < 0.001), 1.7% in institutional homophobia concerning gays (*p* < 0.001), 2.1% in homophobia concerning gays (*p* < 0.001), 1.7% in personal discomfort concerning lesbians (*p* < 0.001), 1.7% in deflection/changeability concerning lesbians (*p* < 0.001), 1.5% in institutional homophobia concerning lesbians (*p* < 0.001), and 1.8% in homophobia concerning lesbians (*p* < 0.001). 

As for the dimension of openness to experiences, it can be seen that this can predict lower personal discomfort and homophobia towards gays. Openness to experiences predicts 0.7% of the variance in the response of the subscale of personal discomfort concerning gays (*p* = 0.015) and 0.5% in homophobia concerning gays (*p* = 0.046); while the dimension conscientiousness/responsibility predicts tolerant attitudes towards feminine sexual orientation and 0.6% of the component of deflection/changeability concerning lesbians (*p* = 0.025). 

## 4. Discussion

Based on the results of the research, it can be seen that, in general, there exists a favourable attitude towards gays and lesbians (Hypothesis 1). Only some students show homophobic attitudes. These results are in the same line as the studies carried out by Serrano et al. [46], Rodríguez [47], and Horne et al. [13], as well as with the conclusions of the World Survey on the Visibility and Public Perception of the LGTBI Collective [48]. The positive influence over the last few decades of legislation concerning equality and discrimination due to reasons of sexual identity and orientation, as well as the work of many organisations concerning awareness-raising, have brought about a significant improvement in the normalisation of sexual diversity [49,50].

Nevertheless, the research shows a slightly greater acceptance of the lesbian collective than of the gay collective. Although the differences in our study are not very significant, other works of research have shown more evident differences in this sense [11,33,51,52], the most favourable attitudes being towards lesbians. In this sense, it has been pointed out that heterosexual men have a more pronounced bias against masculine homosexuality because it deviates from the traditional gender role, influenced by dominant heterosexual norms [53,54] in a western society that still, despite advances, gives greater relevance to the values associated with masculinity [55]. In addition, to justify the more favourable attitudes towards the lesbian collective, some have put forward reasons based on erotic lesbianism that characterises a patriarchal society and masculine culture [27,33,34]. 

Similarly, as the results of our research reflect, young people are more willing to accept institutional policies aimed at protecting gays than those aimed at protecting lesbians. In this sense, Rodríguez et al. [34] found similar results. This may be explained by the greater visibility and victimisation of the gay collective, which thus requires greater legal protection [5].

As for the relation between homophobia and certain personality dimensions (Hypothesis 2), the research demonstrates that extraversion, as a protective factor, and neuroticism, as a risk factor, did not obtain significant correlations. Nevertheless, agreeability is shown to be an essential personality dimension for the rights of the LGTBI collective, as it shows a significant correlation with all the scales of modern homophobia. These results are also apparent in the studies of Horne et al. [13], Miller et al. [14], and Oltra et al. [12]. The young people who show such personality traits as agreeability, cordiality, and social sensibility also show less hostile attitudes towards the gay and lesbian collectives. 

As for the dimension of openness to experiences, the research found a significant correlation with the gay collective but not with the lesbian collective. However, other studies have found that openness to experiences can act as a protective factor against homophobic behaviour patterns towards both collectives [11,56]. 

As for the personality dimension of conscientiousness/responsibility, the research has shown a relationship with the subscale of deflection/changeability in the lesbian collective. This would, therefore, seem to indicate that awareness and social responsibility, as well as a sense of duty, are related to more tolerant attitudes towards lesbian sexual orientation.

As for the relation between moral disengagement and homophobia (Hypothesis 3), the research demonstrates that the greater the level of homophobia, the greater the use of moral disengagement mechanisms by young people will be. The relation is significant on a global scale, as well as in all the subscales of moral disengagement. These results are on the same lines as the studies of Carrera-Fernández et al. [27], Sahlman [28] and Maftei and Holman [57]. The homophobic subjects look for self-justifications for violating the moral standards concerning sexual diversity. They fall back on mechanisms of moral disengagement in situations of direct contact with persons who differ from the heterosexual norms due to perceived discomfort, justifying that this collective can renounce their sexual orientation at will, as well as showing their disagreement with any protective institutional action that may be put forward concerning this collective.

To be precise, the most used mechanisms of moral disengagement are euphemistic language, minimising the responsibility for homophobic attitudes by using words that may be more acceptable; distorting the consequences, ignoring or even distorting negative consequences implied by hostile behaviour towards gays and lesbians; and attributing blame, making both collectives responsible for the hurtful behaviour towards them because of their excessive demands. As pointed out by Rubio-Garay et al. [58] and Bjärehed et al. [25], euphemistic language and distorting consequences are the moral disengagement mechanisms that can best predict aggressive and toxic behaviour patterns. 

Finally, it can be seen that certain personality dimensions and moral disengagement mechanisms act as predictors of the homophobic attitudes of young people (Hypothesis 4). As for personality dimensions, we can conclude that agreeability in the gay and lesbian collectives and openness to experiences in the gay collective act as predictors of homophobia. Thus, as pointed out in other research works [7,10,11,12,13,14], low scores in both of the above predict less tolerant behaviour patterns towards the said collectives, making it more probable that they will violate the rights and freedoms of those who deviate from predominant heterosexual norms. As for moral disengagement mechanisms, it can be seen that they significantly predict homophobic attitudes towards the LGTBI collective. Such results are in line with the studies of Carrera-Fernández et al. [27] and Sahlman [28]. The moral self-control system can be partially set aside in order to violate the rights of the LGTBI collective [18,59].

## 5. Conclusions

Our study has allowed us to demonstrate that, in general, young people have positive, tolerant attitudes towards homosexuality in both its masculine and feminine forms. However, not all young people accept diversity in sexual orientation. The problem currently persists, and the manifestations of rejection and hostility are still present. In this sense, it is particularly relevant to provide data so as to be able to prevent situations of discrimination and harassment suffered by these collectives. 

One important aspect that must be mentioned from our study is that it has provided us with greater knowledge concerning the variables that can predict intimidatory behaviour towards and harassment of the LGTBI collective. Our research has demonstrated that young homophobes can show an apparent acceptance of diversity in sexual orientation, when, in reality, they are uncomfortable in situations in which they are in direct contact with these collectives. They use socially acceptable language to mask their attitudes of intolerance and rejection; they implicitly believe that gays and lesbians can change their sexual orientation at will. Furthermore, they avoid any awareness of the hurt they cause, minimising their morally reprehensible actions. Thus, they fall back on moral disengagement mechanisms to justify their harmful attitudes towards the LGTBI collective. Similarly, it can be seen that low levels of agreeability and openness to experiences predict homophobic behaviour patterns, including moral disengagement to justify their intolerant and intransigent attitudes full of emotional callousness.

Finally, we must point out the fact that this study has its limitations. The data is derived from a very specific context (the university). In addition, the sample is not homogeneous as far as gender is concerned, which could cause a bias when generalising the results. It should also be stressed that, in this research, we did not administer any social desirability scale concerning the participants, which may also be seen as a limitation. 

As for the contribution of the research, we consider that the results are particularly relevant and useful for setting up programmes aimed at preventing, mitigating, and modifying this serious problem of discrimination in the educational sphere. In this sense, we consider that it is crucial to pay attention to the moderating role played by moral disengagement and personality traits in intimidatory and harmful behaviour patterns towards this collective.

We trust that the present research can serve to encourage further study in greater depth.

## Figures and Tables

**Table 1:** Descriptive statistics of the university students in the MHS.

	M	SD	Minimum	Maximum
Personal discomfort (gays)	2.00	1.11	1	5
Deflection/changeability (gays)	1.96	1.03	1	5
Institutional homophobia (gays)	2.12	1.15	1	5
Total homophobia (gays)	1.97	1.09	1	5
Personal discomfort (lesbians)	1.96	1.10	1	5
Deflection/changeability (lesbians)	1.72	0.92	1	5
Institutional homophobia (lesbians)	2.43	1.16	1	5
Total homophobia (lesbians)	1.94	1.05	1	5

**Table 2:** Correlation analysis between moral disengagement, personality dimensions, and modern homophobia.

	Personal Discomfort (Gays)	Deflection/Changeability (Gays)	Institutional Homophobia (Gays)	Total Homophobia (Gays)	Personal Discomfort (Lesbians)	Deflection/Changeability (Lesbians)	Institutional homophobia (Lesbians)	Total Homophobia (Lesbians)
**Moral Disengagement (MD)**								
Moral justification	0.419 ***	0.345 ***	0.407 ***	0.440 ***	0.391 ***	0.301 ***	0.388 ***	0.397 ***
Euphemistic language	0.546 ***	0.444 ***	0.530 ***	0.557 ***	0.541 ***	0.466 ***	0.482 ***	0.557 ***
Advantageous comparison	0.463 ***	0.413 ***	0.482 ***	0.507 ***	0.452 ***	0.412 ***	0.449 ***	0.481 ***
Transfer of responsibility	0.396 ***	0.368 ***	0.394 ***	0.434 ***	0.405 ***	0.393 ***	0.334 ***	0.428 ***
Diffusion of responsibility	0.297 ***	0.275 ***	0.306 ***	0.339 ***	0.291 ***	0.274 ***	0.252 ***	0.295 ***
Distortion of consequences	0.558 ***	0.471 ***	0.569 ***	0.582 ***	0.560 ***	0.511 ***	0.498 ***	0.579 ***
Dehumanisation	0.486 ***	0.385 ***	0.482 ***	0.502 ***	0.460 ***	0.412 ***	0.415 ***	0.479 ***
Attributing blame	0.527 ***	0.456 ***	0.513 ***	0.534 ***	0.491 ***	0.457 ***	0.491 ***	0.530 ***
Total moral disengagement	0.573 ***	0.490 ***	0.572 ***	0.605 ***	0.558 ***	0.497 ***	0.508 ***	0.584 ***
**Personality Dimensions of the (NEO-FFI)**								
Neuroticism	−0.029	−0.017	−0.008	−0.027	−0.022	0.011	0.012	−0.007
Extraversion	0.007	0.001	−0.001	0.004	0.023	−0.050	0.002	0.008
Openness to experiences	−0.084 *	−0.053	−0.038	−0.068 *	−0.058	−0.014	−0.008	−0.043
Agreeability	−0.147 ***	−0.123 ***	−0.131 ***	−0.146 ***	−0.131 ***	−0.129 ***	−0.123 ***	−0.134 ***
Conscientiousness/Responsibility	−0.027	−0.008	−0.009	−0.023	−0.042	−0.077 *	−0.041	−0.052

Note. * *p* < 0.05; *** *p* < 0.001.

**Table 3:** Linear regression of modern homophobia towards the gay collective with respect to moral disengagement and personality dimensions.

	Personal Discomfort (Gays)			Deflection/Changeability (Gays)			Institutional Homophobia (Gays)			Total Homophobia (Gays)		
	R²	β	t	R²	β	t	R²	β	t	R²	β	t
**Moral Disengagement (MD)**												
Moral justification	0.176	0.419	13.430 ***	0.119	0.345	10.705 ***	0.166	0.407	12.974 ***	0.193	0.440	14.245 ***
Euphemistic language	0.298	0.546	18.969 ***	0.197	0.444	14.419 ***	0.281	0.530	18.185 ***	0.311	0.557	19.539 ***
Advantageous comparison	0.214	0.463	15.192 ***	0.171	0.413	13.206 ***	0.232	0.482	15.998 ***	0.257	0.507	17.130 ***
Transfer of responsibility	0.157	0.396	12.560 ***	0.135	0.368	11.511 ***	0.156	0.394	12.494 ***	0.189	0.434	14.034 ***
Diffusion responsibility	0.088	0.297	9.065 ***	0.076	0.275	8.322 ***	0.093	0.306	9.342 ***	0.115	0.339	10.497 ***
Distortion of consequences	0.312	0.558	19.582 ***	0.221	0.471	15.523 ***	0.324	0.569	20.129 ***	0.338	0.582	20.809 ***
Dehumanisation	0.236	0.486	16.168 ***	0.148	0.385	12.151 ***	0.232	0.482	15.992 ***	0.252	0.502	16.894 ***
Attributing blame	0.278	0.527	18.049 ***	0.208	0.456	14.912 ***	0.264	0.513	17.408 ***	0.285	0.534	18.366 ***
Total moral disengagement	0.329	0.573	20.364 ***	0.240	0.490	16.360 ***	0.328	0.572	20.312 ***	0.366	0.605	22.124 ***
**Personality Dimensions (NEO-FFI)**												
Neuroticism	0.001	−0.029	−0.857	0.000	−0.017	−0.497	0.000	−0.008	−0.237	0.001	−0.027	−0.774
Extraversion	0.000	0.007	0.214	0.000	0.001	0.039	0.000	−0.001	−0.022	0.000	0.004	0.112
Openness to experiences	0.007	−0.084	−2.444 *	0.003	−0.053	−1.556	0.001	−0.038	−1.097	0.005	−0.068	−1.996 *
Agreeableness	0.022	−0.147	−4.326 ***	0.015	−0.123	−3.597 ***	0.017	−0.131	−3.859 ***	0.021	−0.146	−4.305 ***
Conscientiousness/Responsibility	0.001	−0.027	−0.774	0.000	−0.008	−0.247	0.000	−0.009	−0.267	0.001	−0.023	−0.669

Note. * *p* < 0.05; *** *p* < 0.001.

**Table 4:** Linear regression of modern homophobia towards the lesbian collective with respect to moral disengagement and personality dimensions.

	Personal Discomfort (Lesbians)			Deflection/Changeability (Lesbians)			Institutional Homophobia (Lesbians)			Total Homophobia (Lesbians)		
	R²	β	t	R²	β	t	R²	β	t	R²	β	t
**Moral Disengagement (MD)**												
Moral justification	0.153	−0.391	−12.379 ***	0.090	−0.301	−9.180 ***	0.151	−0.388	−12.269 ***	0.158	−0.397	−12.606 ***
Euphemistic language	0.293	−0.541	−18.721 ***	0.217	−0.466	−15.320 ***	0.232	−0.482	−16.005 ***	0.310	−0.557	−19.513 ***
Advantageous comparison	0.204	−0.452	−14.754 ***	0.170	−0.412	−13.171 ***	0.202	−0.449	−14.627 ***	0.231	−0.481	−15.968 ***
Transfer of responsibility	0.164	−0.405	−12.889 ***	0.154	−0.393	−12.435 ***	0.111	−0.334	−10.297 ***	0.184	−0.428	−13.799 ***
Diffusion of responsibility	0.085	−0.291	−8.855 ***	0.075	−0.274	−8.305 ***	0.064	−0.252	−7.587 ***	0.087	−0.295	−8.970 ***
Distortion of consequences	0.313	−0.560	−19.650 ***	0.261	−0.511	−17.280 ***	0.248	−0.498	−16.720 ***	0.335	−0.579	−20.668 ***
Dehumanisation	0.212	−0.460	−15.097 ***	0.170	−0.412	−13.169 ***	0.172	−0.415	−13.285 ***	0.230	−0.479	−15.898 ***
Attributing blame	0.241	−0.491	−16.381 ***	0.209	−0.457	−14.947 ***	0.241	−0.491	−16.383 ***	0.280	−0.530	−18.166 ***
Total moral disengagement	0.312	−0.558	−19.579 ***	0.247	−0.497	−16.680 ***	0.258	−0.508	−17.159 ***	0.341	−0.584	−20.952 ***
**Dimensiones de la Personalidad (NEO-FFI)**												
Neuroticism	0.000	0.022	0.644	0.000	−0.011	−0.331	0.000	−0.012	−0.349	0.000	0.007	0.211
Extraversion	0.001	−0.023	−0.655	0.003	0.050	1.464	0.000	−0.002	−0.048	0.000	−0.008	−0.227
Openness to experiences	0.003	0.058	1.705	0.000	0.014	0.412	0.000	0.008	0.241	0.002	0.043	1.241
Agreeability	0.017	0.131	3.835 ***	0.017	0.129	3.777 ***	0.015	0.123	3.592 ***	0.018	0.134	3.925 ***
Conscientiousness/Responsibility	0.002	0.042	1.228	0.006	0.077	2.243*	0.002	0.041	1.195	0.003	0.052	1.505

Note. * *p* < 0.05; *** *p* < 0.001.

## Data Availability

Not applicable.
